# Structure and Properties of Strontium-Modified Zn–Al–Cu Alloys

**DOI:** 10.3390/ma18040797

**Published:** 2025-02-11

**Authors:** Mariusz Krupiński, Krzysztof Labisz, Beata Krupińska

**Affiliations:** 1 Faculty of Mechanical Engineering, Silesian University of Technology, Konarskiego Str. 18a, 44-100 Gliwice, Poland; mariusz.krupinski@polsl.pl; 2 Faculty of Transport and Aviation Engineering, Silesian University of Technology, Krasińskiego Str. 8, 40-019 Katowice, Poland

**Keywords:** Zn alloys, alkaline earth metals, crystallization kinetics, structure, abrasive wear

## Abstract

(1) In recent years, an increase in the available functionality of non-ferrous alloys has been observed based on the modification and optimization of their chemical composition. This study investigated the effect of Sr addition on the structure and properties of hypereutectic Zn–Al–Cu alloys. The objective was to determine how a modification with Al–Sr master alloy affects the crystallization kinetics, microstructure, hardness, and abrasive wear resistance and whether the modification of the phase composition reduces the corrosion resistance. (2) The total influence of strontium was determined based on the microstructure, phase composition, and derivative curve changes of the tested Zn–Al–Cu alloys with added Sr. Optical microscopy, scanning electron microscopy (SEM), and transmission electron microscopy (TEM) were used to analyze the influence of chemical and phase composition, and thermo-derivative analysis (TDA) was used to investigate the crystallization kinetics of zinc alloys with different chemical compositions. (3) Sr modification caused the formation of primary Al_2_Sr phases in the Zn alloy and also secondary Zn_13_Sr and Al_4_Sr phases (depending on the melting temperature of the alloy). (4) The primary and secondary intermetallic phases with strontium increased the hardness by approx. 20% and the abrasion resistance by approx. 7.5%.

## 1. Introduction

Zinc alloys are used for the mass production of components with high dimensional accuracy. They are characterized by the possibility of using them at a higher casting speed, while ensuring increased durability of the die, which predisposes them to large-scale production of small castings. Zinc alloy castings are widely used in the automotive and electronics industries as well as in mechanical engineering [1].

In order to improve the mechanical properties, including the hardness and abrasion resistance, of zinc casting alloys, the procedure of modifying the casting structure is used. This involves changing the morphology of the structural components of the alloy, primarily by reducing the interfacial distance of the α′ + η eutectic, fragmenting the components’ structures, and (as in the case of La and Ce) changing the morphology of the α′ phase [2,3].

An important factor leading to an improvement in the quality of cast products is the appropriate use of knowledge about crystallization and its mechanisms, which allows the production of castings with optimal microstructure and properties. In the case of casting alloys, the crystallization process takes place over a temperature range determined by the values for the beginning and end of crystallization, i.e., between the liquidus and solidus temperatures, which depend primarily on the alloy composition, cooling rate, and the thermodynamic conditions of transformation. The free energy values for the liquid and solid phases depend on the concentration of the remaining components in the liquid phase [4,5,6].

The main zinc alloying elements are Cu, which influences the formation of the hexagonal ε phase (Zn_4_Cu) and the hexagonal τ phase (Zn_3_Cu intermetallic compound). The τ phase crystallizes at a concentration range of 0.6–0.7% Cu in the alloy and causes dimensional changes in the castings [7]. Moreover, the addition of Ti causes fragmentation of α-phase dendrites due to heterogeneous nucleation of this phase on Al_3_Ti and Zn_3_Ti secretions [8,9].

ZnAl_25_ alloys require a melting point of about 700–750 °C, which can cause the alloy to oxidize and can lead to increasing energy costs. A newly developed master added in the melting process shows good solubility at much lower temperatures, i.e., about 500 °C in Zn–Al alloys, thus avoiding harmful overheating and saving energy costs [10]. The casting temperature also has a significant impact on the mechanical properties of zinc castings, which has also been confirmed by the authors of [11]. The best tensile strength values were achieved for Zn–Al alloys with 4.6 wt.% Al, for a soaking temperature of 460 °C before alloy casting.

Strontium is a frequently used modifier of the microstructure of metal alloys because it is a long-term modifier, its effects persisting even after many melts of the alloy [12,13]. Additionally, the Sr used as a modifier for Zn–Al alloys does not negatively affect corrosion resistance, which has been confirmed by previous test results [14].

Ref. [15] investigated the effect of heat treatment of ZA-27 alloys on sliding wear behavior. The heat treatment caused a decrease in hardness and tensile strength, but also an increase in elongation. The heat-treated alloy samples achieved higher tribological behavior compared with the cast samples, both in terms of friction and wear resistance. The improved tribological behavior of the heat-treated alloys, despite their reduced hardness, may be the result of breaks and discontinuities in the dendritic structure. Dendritic segregation influences the phenomena involved in the structural transformations occurring both during solidification and cooling in the solid state [16].

This investigation describes the effect of strontium modification on the following:

- the crystallization kinetics of the hypereutectic Zn–Al–Cu alloy solidifying between temperatures of 450 °C and 700 °C,
- the formation and distribution of Sr-containing intermetallic phases,
- the influence of the microstructure on the mechanical properties and corrosion resistance.

## 2. Materials and Methods

The tests were performed on laboratory-produced alloys to which appropriate amounts of Al and Cu alloying elements and an Al–Sr master alloy were introduced (with 10% mass concentration of Sr). The mass concentration of aluminum was set to obtain hypereutectic alloys, i.e., in a range from 8 to 12%. The copper content was kept in a range of about 1 wt.%. The melts were made in a resistance furnace in chamotte-graphite crucibles, using a protective argon atmosphere. The manufactured alloys were cast into properly prepared metal crucibles made of carbon steel. The control tests of the mass concentrations of the Al, Cu, and Sr alloy additives were carried out by ICP-OES with a Jobin-Yvon-Horiba ULTIMA 2 device. The chemical composition of the obtained alloys is given in Table 1.

Thermal analysis of the tested alloys was performed on the UMSA MT5 device, patent no. 60/339,358. From the previously produced casts, samples for thermal tests were prepared, with dimensions of ø30 × 35 mm, which were then melted in a graphite crucible and smeared with a boron nitride suspension. Type K thermocouples were each placed in the same place (in the heat node), the position of which was determined during the preliminary tests.

Metallographic examinations were performed using a Zeiss Axio Observer metallographic microscope (SEM, Thornwood, NY, USA) employing a computer image analysis system at magnifications from 50 to 500 times and an Olympus LEXT OLS4000 confocal microscope (Tokyo, Japan). The prepared specimens were etched in 10% HF. The etching time and the concentration of the reagent were experimentally selected for each tested alloy.

Qualitative and quantitative X-ray microanalysis, as well as the analysis of the surface distribution of the alloying elements, were performed on specimens in a direction transverse to the casting direction using an Oxford LINK EDS ISIS X-ray spectrometer (EDS, Oxford, Oxford Instruments, Abingdon, UK) at an accelerating voltage of 15 kV and a JEOL JXA 733 X-ray microanalyzer (Oxford, Oxford Instruments, Abingdon, UK).

Microstructural investigations of thin foils and phase identification of the precipitates were performed using a JEOL 3010CX TEM (FEI Company, Hillsboro, OR, USA), at an accelerating voltage of 300 kV, with the use of the selected area diffraction (SAD) technique, allowing for the identification of phase components. The resolution allows it to very accurately determine the phases occurring in this alloy or in even smaller structural components [17]. The obtained diffractograms were analyzed using specialized software dedicated to solving electron diffractograms. The procedure for making thin films consisted of cutting thin plaques from the material to be tested, then grinding these to a maximum thickness of 80 μm, and cutting disks with a diameter of 3 mm. The cut discs were then polished using a Gatan 691 (PIPS ™, Pleasanton, CA, USA) ion polishing system until one or more holes were visible. Ion polishing was carried out using an ionized argon beam at a voltage of 3.5 kV, directed at an angle of 3.5–4.5° to the sample surface.

In order to determine the resistance to erosive wear of Zn–Al–Cu alloys after the specified modifications, tests were performed in accordance with ASTM G 76–95 (the standard test method for conducting erosion tests by solid particle impingement) [18]. The device was calibrated prior to the tests. The results showed that the mass loss of the reference sample complied with the conditions specified in the standard. The tests of resistance to erosive wear were carried out for a test duration of 10 min and set erodent (Al_2_O_3_) incidence angles of 90°.

The abrasion resistance tests were carried out using a tribometer (ball on plate), in which a steel ball with a diameter of 6 mm made a reciprocating movement on the surface of the tested samples with a maximum surface roughness of Ra 0.1 at a speed of 40 mms^−1^ and a pressure of 10 N. The total friction path was 50 m.

The hardness was measured using a Zwick ZHR 4150 hardness tester (WroclawPoland, Europe), using the Rockwell hardness method on the HRA scale. To obtain a correct result, at least 10 measurement points were performed for each measurement, and a statistical analysis of the obtained results was performed.

Corrosion resistance tests (for 18 samples) were carried out in a salt spray chamber CC450iP—Ascott (Ascott, Singapore) according to the guidelines contained in ISO 9227: 2006: Corrosion tests in artificial atmospheres—Salt spray tests [19]. The samples were weighed before testing. The standard salt spray corrosion resistance test consisted of the following steps: preparation of a 5% brine solution; placing the samples at a 15-degree angle in the salt chamber; starting the test; taking photographs after 2, 6, 24, 48, and 96 h; drying the samples after the test; rinsing the samples with running water; and drying again with a stream of air. The samples were then cleaned of corrosion products in glycine.

Before testing, the samples were weighed, placed in a salt chamber (at an angle of 15°), and treated with a 5% brine solution for several dozen hours. After testing, the samples were rinsed with a stream of water, rinsed in aqueous glycine according to the standard, and weighed to confirm the removal of the corrosion products. The mass loss of the sample V_c_ (1) was determined from the dependence V_p_ (2), which revealed the linear corrosion rate described by the relationship in Equation (1):

V_c_ = Δ_mk_ · (A·t_k_)^−1^ (1)

V_p_ = (V_c_ · 365) · (1000 · d)^−1^ (2)

A description of the designations in the formulas is as follows:
V_c_—loss of mass/g · (m^2^ · day)^−1^,
∆_mk_—sample mass change/g,
A—active sample surface against corrosion/m^2^,
t_k_—duration of corrosion/day,
V_p_—linear corrosion rate/mm · year^−1^,
d—material density/g · (cm^3^)^−1^.

## 3. Results and Discussion

### 3.1. Thermo-Derivative Analysis (TDA)

The driving force of crystallization in the case of alloys is the difference between the free energy of the liquid and the energy of the mixture of liquid and solid phases over the range of the concentration of the second component for the liquid and solid phases. It was therefore found useful, both from a practical and theoretical point of view, to learn more about the influence of Sr addition on the crystallization kinetics and thus the microstructural and mechanical properties of the ZnAl8Cu1Sr1 alloy.

**Figure 1:**
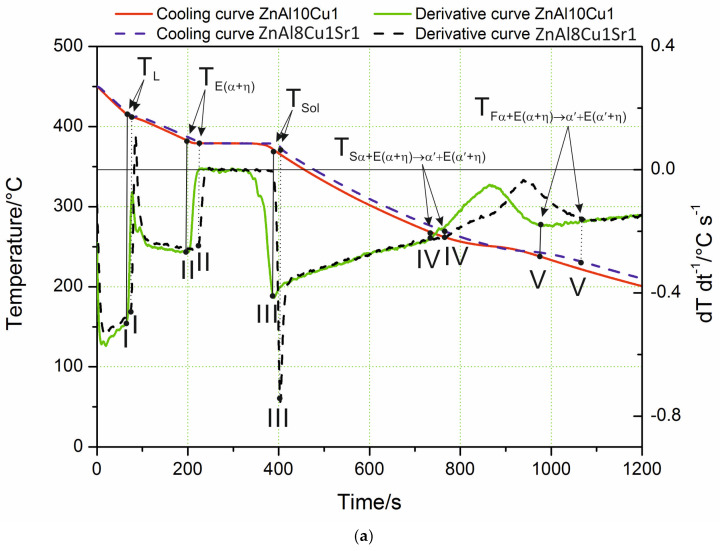

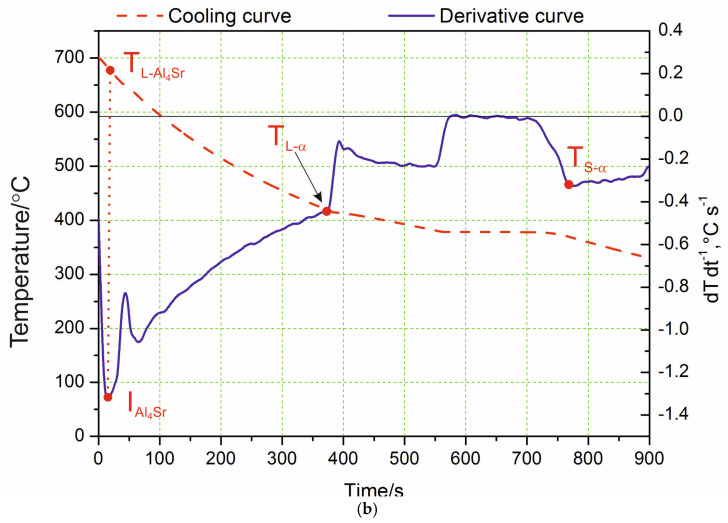
(**a**) Cooling curve and derivative curve of the tested alloys cooled at a rate of 0.1 °C∙s^−1^ from a temperature of 450 °C (Table 2). (**b**) Cooling curve and derivative curve of the tested alloys cooled at a rate of 0.1 °C∙s^−1^ from a temperature of 700 °C (Table 2).

The characteristic cooling curve and derivative curve for ZnAl8Cu1Sr1 as-cast alloys cooling freely at a rate of 0.1 °C∙s^−1^ are shown in Figure 1a. Analyzing the course of the crystallization process based on the curves obtained, it could be concluded that the alloy did not show significant changes in the stages of the crystallization of individual components in comparison with the crystallization effects recorded for the ZnAl10Cu1 alloy.

Analyzing the course of the crystallization process based on the obtained curves of changes in temperature and the dT·dt^−1^ derivative, it was found that the process of α-phase nucleation began at the T_L_ temperature, which is illustrated on the derivative curve in the form of a slight inflection at point I and a temporary decrease in the cooling rate of the alloy. The thermal effects accompanying the process of nucleation and the growth of α crystals provided additional heat to the mixture of liquid and solid phase being formed; however, the heat balance of the cooling ingot was negative. The chemical composition of the remaining liquid changed according to the liquidus line of the Zn–Al diagram, which was also confirmed by the studies in [20]. In this range, an approximately constant negative value of the dT·dt^−1^ derivative was observed. The liquid became increasingly enriched with zinc, and, after reaching the temperature T_(Al+Zn)_, nucleation of the α + η eutectic took place. As a result of further cooling, the remaining liquid theoretically crystallized at a constant temperature, T_E(Al+Zn)_ (dT·dt^−1^ = 0), creating the α + η eutectic (point II). Crystallization ended when the melt reached the temperature T_Sol_ (Figure 1a). In experimental practice, the recording of temperatures characteristic of the beginning and end of the eutectic transformation showed a slight difference; it was assumed that the solidus temperature T_Sol_ was the temperature value corresponding to point III (end of eutectic solidification). The differences become visible when the melting temperature of the liquid alloy increased to 700 °C. The crystallization then started from the Al_4_Sr phase at 657 °C (Figure 1b). Only at the next point did the α phase crystallize. Table 2 shows the reactions that occurred in the tested alloys during solidification and in the solid state, as well as the temperature at characteristic points. Data are provided for both the alloy with the addition of strontium and the reference alloy without any Sr. There were no differences in the crystallization temperature of the α phase and α + η eutectic caused by the difference in the mass concentration of Al and the addition of Sr to the alloy.

**Figure 2:**
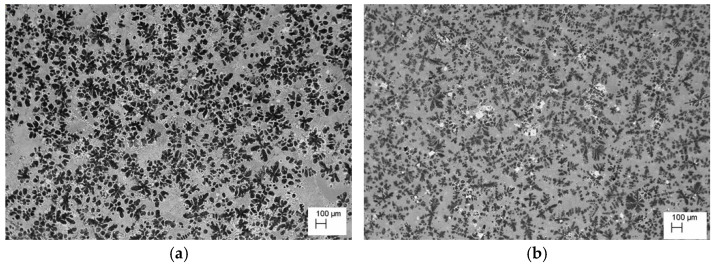
Microstructure of the alloy cooling freely with a rate 0.1 °C∙s^−1^. (**a**) ZnAl10Cu1, (**b**) ZnAl8Cu1Sr1.

### 3.2. Microstructure and Phase Composition

**Figure 3:**
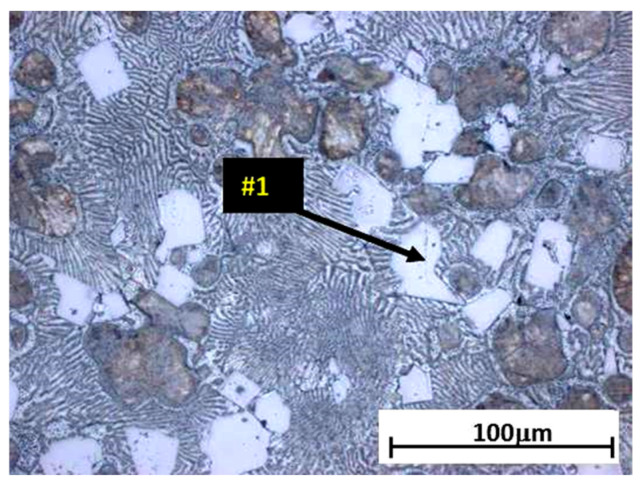
Alloy microstructure of ZnAl8Cu1Sr1, cooling rate 0.1 °C∙s^−1^, #1—Al_2_Sr.

**Figure 4:**
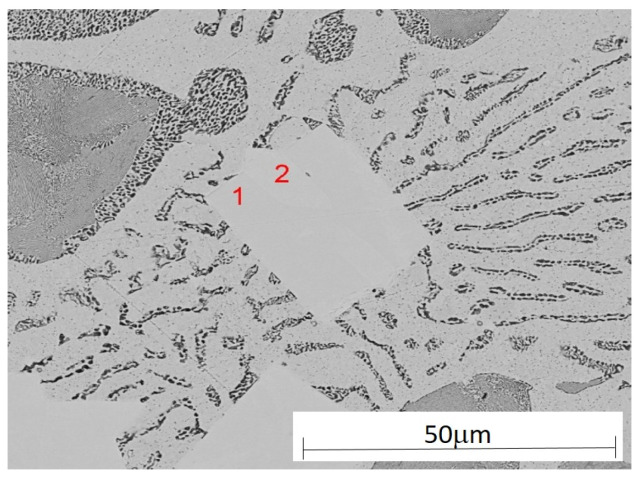
Microstructure of the ZnAl8Cu1Sr1 alloy with marked Sr phases (point 1 and 2 on Figure 4) with the performed wavelength dispersive X-ray spectroscopy (WDS) analysis (Table 3).

The analysis of the effects of phase transformations during the crystallization of alloys was supplemented with structural studies. The microstructure of the ZnAl10Cu1 alloy is shown in Figure 2a. The structural components were the α′ phase and the α′ + η eutectic. The metallographic analyses of the strontium-modified Zn–Al–Cu alloys confirmed the presence of the α′ phase and the “fine eutectic” formed during the monotectoid transformation, as well as the α′ + η eutectic (Figure 2b). Moreover, optical microscopy tests revealed the Al_2_Sr phase in the form of partially quasi-spherical and/or rectangular precipitates with straight edges (Figure 3 and Figure 4, Table 3).

The Al_2_Sr intermetallic phase occurred in the alloy, having a melting point of 928 °C [21]. The Al_2_Sr phase was shown to be the primary phase because there were no visible thermal effects on the derivative curve for the tested alloys, both at the melting point of 450 °C and at 700 °C. The Al_2_Sr phase, with an average microhardness of 400.54 HV_0.01,_ was also responsible for the increase in hardness and abrasion resistance of the tested alloys. In the alloy solidified from a temperature of 700 °C, the Al_4_Sr [12] phase crystallized at a temperature of 657 °C; this phase was a structural component of the investigated material. Part of the strontium addition went into solution even at 450 °C and formed Zn_13_Sr phases during solidification. Similar results were obtained by researchers in [22].

**Figure 5:**
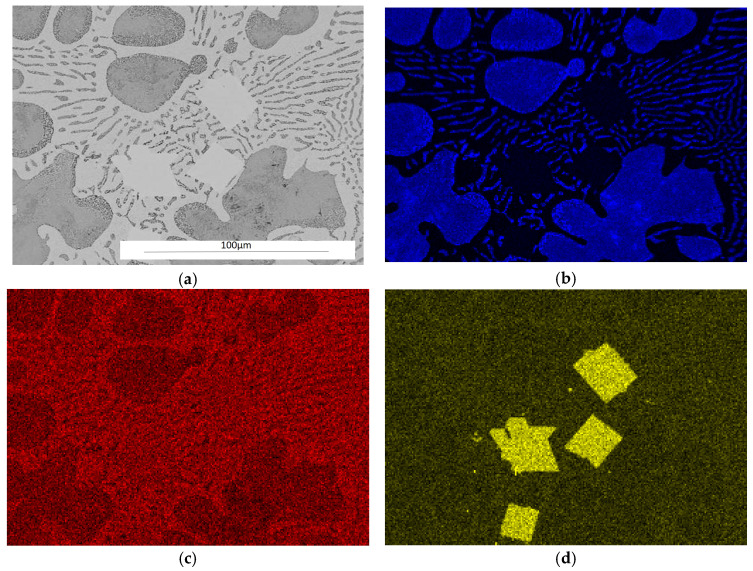
Microstructure of the ZnAl8Cu1Sr1 alloy cooled with a rate of 0.1 °C∙s^−1^, secondary electron TEM image (SE) as well as elemental mapping: (**a**) SE; (**b**) Al; (**c**) Zn; (**d**) Sr.

Figure 5 shows the surface distribution of the elements that are components of the ZnAl8Cu1Sr1 alloy structure.

**Figure 6:**
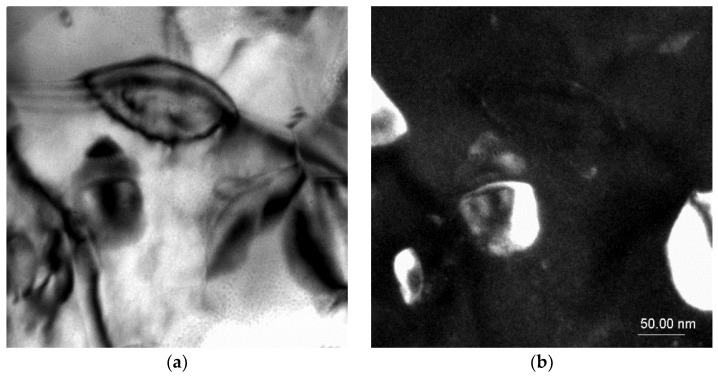

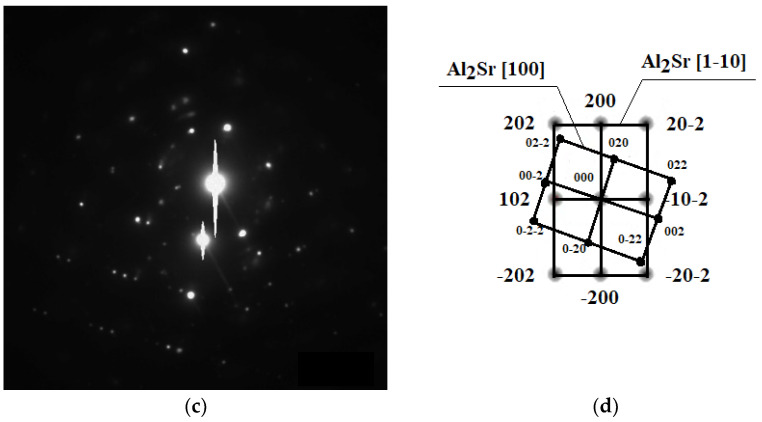
TEM microstructure of the investigated alloys. (**a**) Bright field image of the Al_2_Sr phase, (**b**) dark field image of the Al_2_Sr particle, (**c**) diffraction pattern of the determined phase, and (**d**) solution of the diffraction pattern presented in (**c**).

TEM investigations confirmed the presence of intermetallic strontium phases, especially of the Al_2_Sr phase present in the eutectic; the noted phase particle had a zone axis of [100] as well as [1–10] (Figure 6). The morphologies of these phases were relatively similar to each other, the particles having a bulk globular shape with a size of ca. 5 to 100 μm.

**Figure 7:**
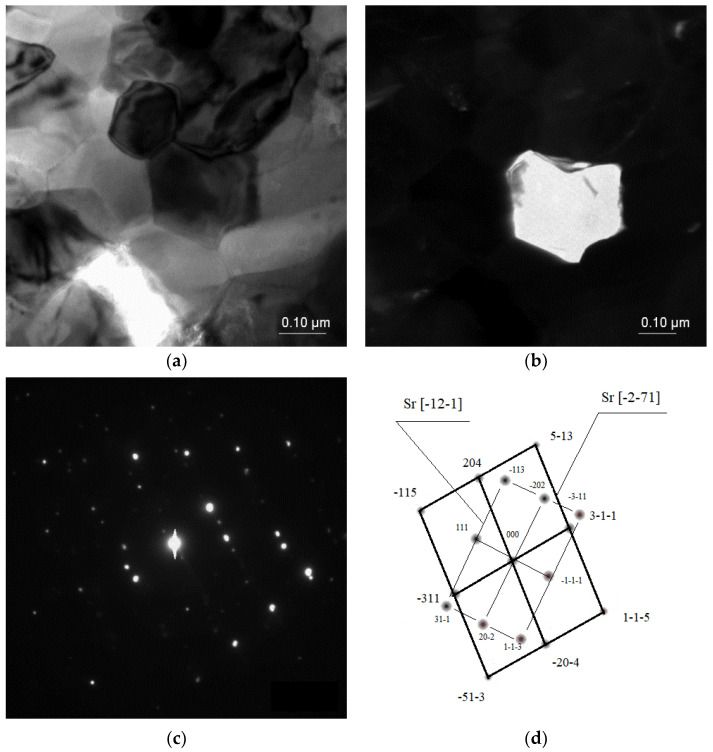
TEM microstructure of the investigated alloys. (**a**) Bright field image of the Sr phase, (**b**) dark field image of the Sr particle, (**c**) diffraction pattern of the determined phases, and (**d**) solution of the diffraction pattern presented in (**c**).

In some cases, the Sr phase particles were discovered with zone axes of [−12–1] and [−2–71], with a size up to 40 μm, and with more sharp edges compared with the Al_2_Sr phase particles (Figure 7).

### 3.3. Erosion Wear Resistance Results

**Figure 8:**
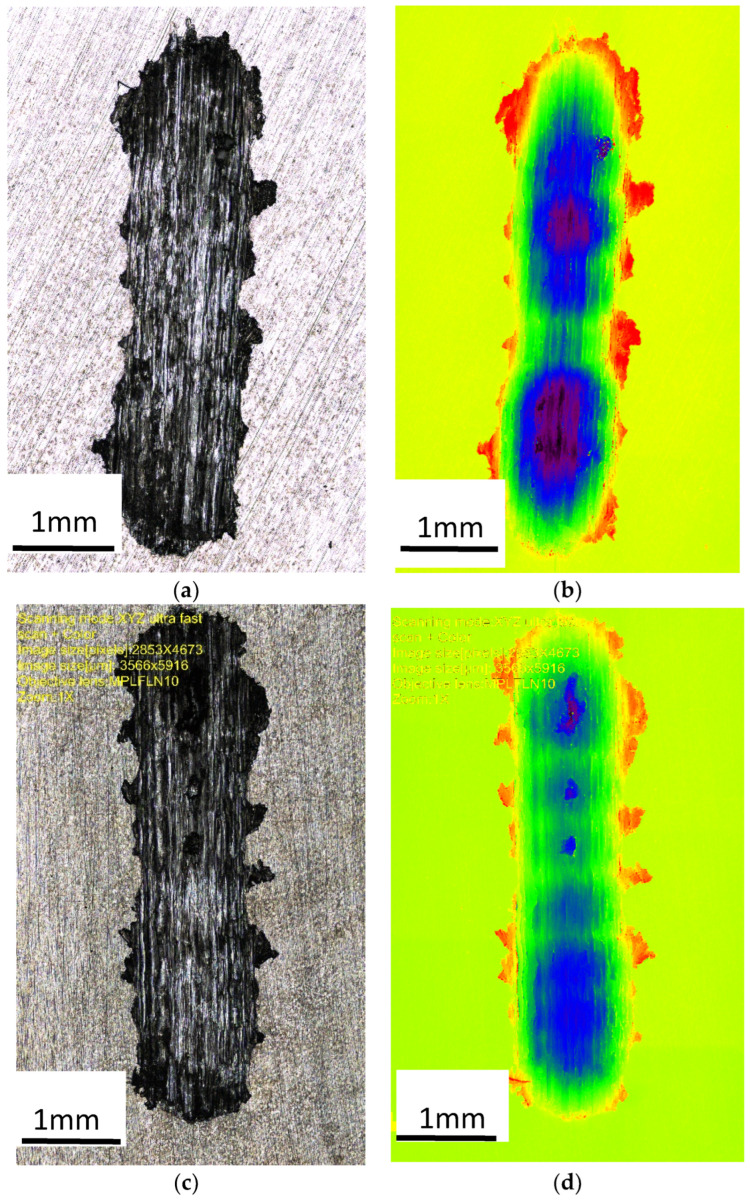
Image of the wear trace after the ball-on-plate test for the alloy cooled at a rate of 1 °C∙s^−1^. (**a**) ZnAl10Cu1—wipe; (**b**) ZnAl10Cu1—image in a color scale; (**c**) ZnAl8Cu1Sr1—wipe; (**d**) ZnAl8Cu1Sr1—image in a color scale.

Figure 8 shows an image of the abrasion observed with a confocal microscope, which provided the possibility of assessing the height of the abrasion, measuring its depth, and presenting the surface (in a color scale).

The abrasion was clear in the image captured by a confocal microscope (Figure 8), which provided high measurement resolution and allowed evaluations of the height in the surface images (using a color scale). The accumulation of material on the surface of the test sample (red color) and the indentations (dark blue color) were created as a result of the reciprocating movement of the ball; the ZnAl8Cu1Sr1 alloy was more evenly worn over the entire friction path. This indicated increased uniformity in terms of the distribution of fine-grained structural components and related properties.

The presence of many primary phases in the microstructure led to a more significant increase in abrasion resistance, as observed following modifications with strontium Al_2_Sr, Al_4_Sr, and Zn_13_Sr. The wear area was 7.5% lower compared with the alloy without Sr addition (Table 4, Figure 12). This difference was caused by the uniform distribution of the primary phases Al_2_Sr and secondary phases Al_4_Sr, in the α′ + η. eutectic. Based on the obtained results of the wear trace tests, typical wear features characteristic of abrasive wear were noted, including grooves, but also dents and plastic deformation, indicating material displacement during the abrasion resistance test.

**Figure 9:**
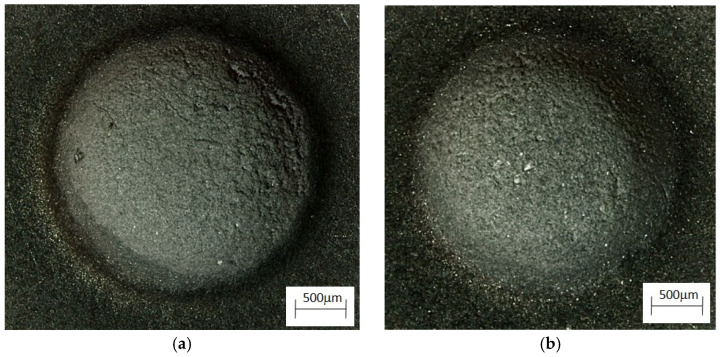
Alloys after erosion test at an angle of incidence of the erodent of 90°. (**a**) ZnAl10Cu1; (**b**) ZnAl8Cu1Sr1.

By introducing additional alloying elements (modifiers) to the zinc, changes in the physical properties of the material, such as resistance to abrasion, but also changes in the effects of the erosive action of the environment, were expected. For this reason, research was undertaken to assess the surface erosivity of the tested alloys (Figure 9). A stream of Al_2_O_3_ was directed onto the sample surface. The test results clearly indicated an increase in the erosive wear resistance of strontium-modified alloys. The mass loss after erosion test for the ZnAl10Cu1 alloy is 0.024 g, and for the ZnAl8Cu1Sr1 alloy is 0.016 g. The mass loss results from the erosion resistance test are presented in a summary graph (Figure 12).

### 3.4. Hardness Results

The increased uniformity of the distribution of hard, fine-grained structural components and the related mechanical properties was the reason for the increased HRA hardness of the tested alloys (Table 5, Figure 12).

The presence of many intermetallic phases in the microstructure led to a more significant increase in abrasion resistance and hardness, as observed following modifications with strontium. Therefore, the influence of the modifier (i.e., Sr) on the corrosion resistance was also investigated (Section 3.5).

### 3.5. Corrosion Results

**Figure 10:**
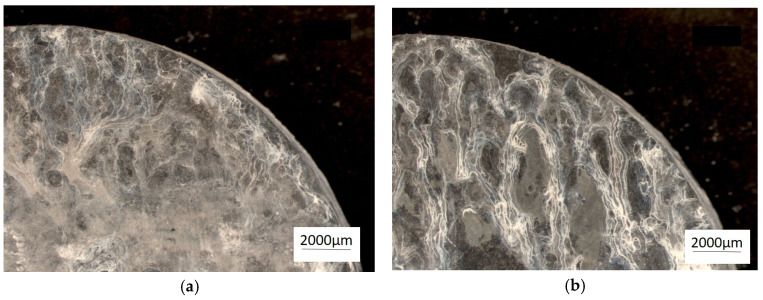
Chosen samples after corrosion test in a salt mist atmosphere. (**a**) ZnAl10Cu1 and (**b**) ZnAl8Cu1Sr1.

The mass losses recorded for the unmodified and Sr-modified alloys were very similar: ZnAl10Cu1 alloy = 0.084 g∙(m^2^∙day)^−1^ and ZnAl8Cu1Sr1 alloy = 0.077 g∙(m^2^∙day)^−1^ (Figure 10).

**Figure 11:**
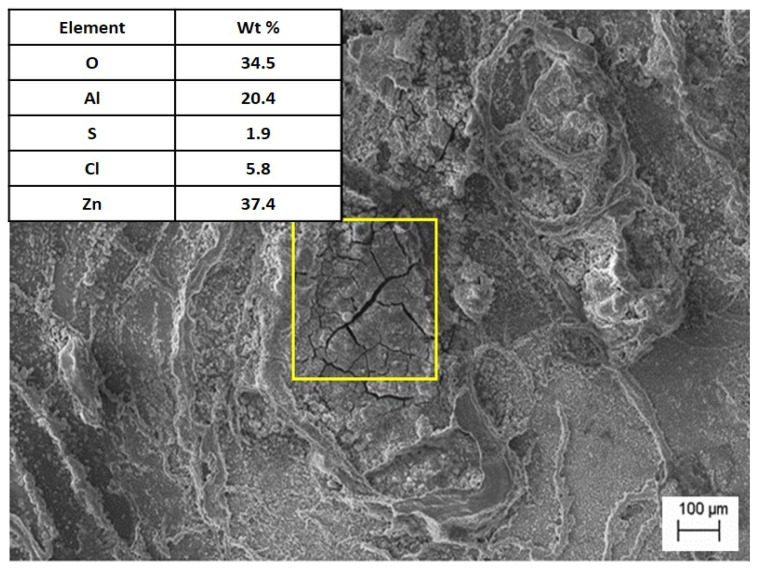
The microstructure of the alloy after the corrosion test with EDS analysis in the marked area.

Corrosion tests in salt water indicated that the presence of Al in Zn–Al alloys can hinder passivation. This was also confirmed by other researchers in their studies [22]. A salt spray corrosion test revealed that the Sr-modified alloy, cooling freely at a rate of 0.1 °C∙s^−1^, exhibited a mass loss comparable to that of unmodified alloys. The occurrence of lamellar eutectic, in which both phases are mutually separated, reduces the rate of corrosion processes, in contrast to the precipitation of intermetallic phases. In the studies, the occurrence of intergranular corrosion was observed, which is confirmed by other researchers [22]. Tests showed that the products of corrosion, apart from oxides, were zinc chloride (Figure 11). This behavior resulted from the smaller specific surface of the grain boundaries [23], as well as the formation of intermetallic phases containing Sr, which reduced the cathodic reduction of oxygen and the formation of a passive layer.

**Figure 12:**
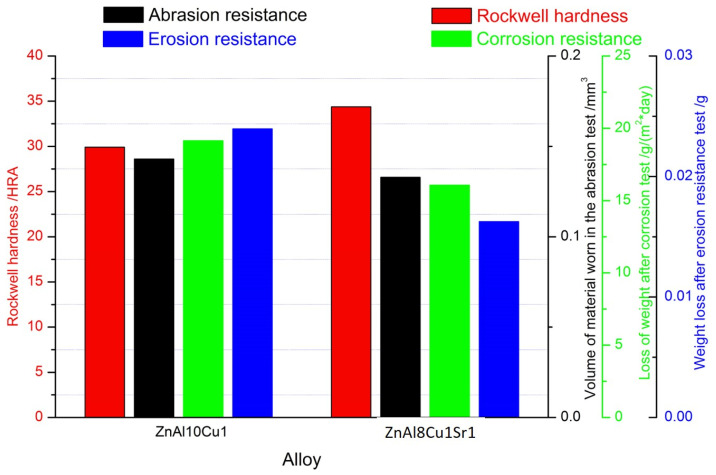
The properties of tested alloys: ZnAl10Cu1 and ZnAl8Cu1Sr1.

Figure 12 compares the tested properties and corrosion resistance of ZnAl10Cu1 and ZnAl8Cu1Sr1 alloys. The graph shows an increase in the hardness of the alloy with the addition of Sr, with a simultaneous decrease in the wear surface measured both in the ball-on-plate test and in the erosion resistance test.

## 4. Conclusions

The conducted studies showed that the modification of the chemical composition with the Al–Sr master alloy increased the resistance to abrasive wear and erosion, as mentioned in [24], but not as a result of the modification of the structural components of the alloy. In multicomponent systems, the exact values of the start and end temperatures of the solidification of structural components can be determined using TDA, and these values can differ significantly compared with two-component systems.

In general, the following can be stated:

- in Zn–Al–Cu alloys modified with Sr, the structural components, i.e., α′ and the α′ + η eutectic, were not modified, and the temperature of the beginning and end of crystallization of the phases and eutectic did not change either.
- the change in the mass concentration of Al (in the range from 8 to 10 wt.%) in eutectic Zn–Al–Cu alloys did not change the start and end temperatures of solidification of the α′ phase and α′ + η eutectic. It changed the area between the derivation and calorimetric curves, which changed the share of individual phases.
- the improvement in mechanical properties occurred as a result of the presence of the primary Al_2_Sr and secondary Al_4_Sr phases (in alloys melted at 700 °C), as well as intermetallic phases (Zn_13_Sr).
- in the alloy structure, in addition to the aluminum–strontium phases, there were also pure Sr phases. These hard intermetallic phases improved the mechanical properties, while not worsening the resistance to corrosion.
- the difference in corrosion resistance measured according to the standard ISO 9227: 2006: (Corrosion tests in artificial atmospheres—Salt spray tests) [19] did not exceed 9%, the alloy with Sr being more resistant.
- the addition of Sr also caused a shift in the end temperature of the monotectoid transformation T_Fα+E(α+η)→α′+E(α′+η)_ by about 12 °C and also caused a shift in time toward higher values compared with the alloy without the addition of strontium.

## Figures and Tables

**Table 1:** Chemical composition of the investigated alloys.

Alloy	Alloy Description	Mass Concentration of Alloying Elements, %			
		Al	Cu	Sr	Zn
1	ZnAl8Cu1Sr1	8.51	0.93	1.02	balance
2	ZnAl10Cu1	10.02	0.98	-	balance

**Table 2:** Crystallization temperature of phases and eutectics, as well as the beginning and end of monotectoid transformation.

Markings on Figure 2a,b	Reaction		Temperature, °C	
	ZnAl8Cu1Sr1	ZnAl10Cu1	ZnAl8Cu1Sr1	ZnAl10Cu1
I_Al4Sr_	L → Al_4_Sr	-	657	-
I	L → α	L → α	415	415
II	L → E_(α+η)_	L → E_(α+η)_	411	410
III	L → Sol	L → Sol	372	370
IV	S_α+E(α+η)→α′+E(α′+η)_	S_α+E(α+η)→α′+E(α′+η)_	273	270
V	F_α+E(α+η)→α′+E(α′+η)_	F_α+E(α+η)→α′+E(α′+η)_	224	236

**Table 3:** The results of the quantitative WDS analysis of the chemical composition of the ZnAl8Cu1Sr1 alloy, performed in the places marked in Figure 4.

Analysis Point	Mass Concentration of the Element, %			
	Al	Sr	Cu	Zn
1	5.3	23.6	3.2	67.9
2	5.7	25.3	2.1	66.9

**Table 4:** Value of the volume of the wear trace of the tested alloys.

Alloy	The Volume of Material Worn in the Abrasion Test	Volume Difference Compared with the Sample Without Modification	
	mm^3^	mm^3^	%
ZnAl10Cu1	0.14	0	0
ZnAl8Cu1Sr1	0.13	−0.01	−7.4

**Table 5:** Statistical analysis of the hardness measurements of the tested alloys.

Alloy	Hardness Mean Value, HRA	Standard Deviation	Change of the Average Hardness Value, %
ZnAl10Cu1	29.91	1.43	0.00
ZnAl8Cu1Sr1	35.29	2.38	17.97

## Data Availability

The original contributions presented in this study are included in the article. Further inquiries can be directed to the corresponding author(s).
